# Multitarget Actions of Pentacyclic Triterpenic Acids in Alzheimer’s Disease: Mechanistic Insights

**DOI:** 10.3390/molecules31122018

**Published:** 2026-06-09

**Authors:** Niti Sharma, Seong Soo A. An

**Affiliations:** Lab of Ageing-Related Diseases, Bionano Research Institute, Gachon University, 1342 Seongnam-daero, Sujung-gu, Seongnam-si 461-701, Gyeonggi-do, Republic of Korea; nitisharma@gachon.ac.kr

**Keywords:** Alzheimer’s disease, pentacyclic triterpenic acids, neuroprotection, multitarget therapy, mechanism of action, phytochemicals

## Abstract

Alzheimer’s disease (AD) is a complex neurodegenerative disorder with features of amyloid-beta (Aβ) accumulations, tau hyperphosphorylation, oxidative stress, neuroinflammation, and synaptic losses. Despite extensive therapeutic investigations for many decades, the clinical treatment options remained largely symptomatic, while anti-amyloid antibody therapies were expensive and had limited accessibility. A subclass of triterpenoids generated from plants, pentacyclic triterpenic acids (PTAs), exhibited a variety of pharmacological properties. The neuroprotective effects of some important PTAs in AD models were reviewed in this study. These phytochemicals displayed a multimodal neuroprotection by lowering amyloid and tau, improving mitochondrial function, inhibiting inflammation, and improving synaptic plasticity and cognition. However, the neuroprotective mechanisms of several PTAs remained poorly characterized. In addition, most evidence were preclinical, while poor bioavailability and the limited clinical validation hindered the therapeutic translation. Studies were needed to evaluate these phytochemicals in AD, improve their pharmacokinetics, and enhance brain delivery. Their diverse bioactivities and encouraging preclinical findings suggest these compounds may serve as promising lead candidates for future drug development in neurodegenerative diseases.

## 1. Introduction

Alzheimer’s disease (AD) is the leading cause of dementia worldwide. It was characterized by amyloid-beta (Aβ) deposition, tau protein hyperphosphorylation, oxidative stress, mitochondrial impairment, chronic neuroinflammation, and synaptic loss [1,2]. These overlapping mechanisms collectively contribute to progressive neuronal degeneration and cognitive decline. The existing treatments primarily provide symptomatic relief rather than modifying the disease itself. These mainly include cholinesterase inhibitors and memantine for symptom management. New antibody therapies, such as lecanemab and donanemab, targeted amyloid plaques and may slow disease progression. However, these therapies were costly and could cause significant side effects. These limitations increased interest in multitarget therapeutic approaches, particularly low-cost, naturally derived compounds with broad neuroprotective properties [3]. AD pathophysiology involved multiple interconnected pathological pathways, and single-target therapies often showed limited clinical benefit. This increased interest in multitarget therapeutic (or multitarget-direct ligands: MTDLs) approaches, particularly naturally derived compounds capable of simultaneously modulating oxidative stress, neuroinflammation, protein aggregation, mitochondrial dysfunction, and synaptic impairment. Several phytochemicals demonstrate the ability to protect neurons by modulating multiple pathological pathways and improving memory and cognition [4].

Pentacyclic triterpenic acids (PTAs), a subclass of plant-derived triterpenoids, attracted attention because of their wide range of therapeutic activities [5,6,7,8,9,10]. Triterpenoids were a large and diverse class of natural compounds composed of 30 carbon (C30) atoms, typically derived from six isoprene (C_5_H_8_) units. PTAs were oxidized triterpenoids that contained one or more carboxylic acid groups (-COOH). They were typically derived from pentacyclic triterpenes, such as oleanane, ursane, and lupane types. PTAs shared many pharmacological properties with other triterpenoids but tended to exhibit stronger activity due to increased polarity (from the -COOH group) and better interactions with the membrane proteins and active sites of the enzymes [11,12,13]. Although MTDLs offer broader therapeutic coverage, challenges including pharmacokinetic complexity, off-target effects, and BBB delivery remain important limitations. These physicochemical features may also influence BBB permeability, as effective central nervous system (CNS) accumulation depended not only on lipophilicity and passive diffusion but also on efflux transporters, such as P-glycoprotein (P-gp) and breast cancer resistance protein (BCRP).

In our previous review, the important role of tetracyclic triterpenoids in neuroprotection was discussed, owing to their ability to traverse the blood–brain barrier (BBB) [5]. As a follow-up, the mechanisms of neuroprotection by several important PTAs, namely asiatic acid, betulinic acid, boswellic acid, maslinic acid, oleanolic acid, ursolic acid, and glycyrrhetinic acid, in AD were presented in the present review. These compounds exhibit multimodal neuroprotective actions, including antioxidant, anti-inflammatory, anti-amyloidogenic, and synaptic plasticity-enhancing effects. Several of them were also associated with the regulation of tau-related pathways, autophagy, and proteostasis. They modulate key AD-related pathways, including nuclear factor erythroid 2-related factor 2/antioxidant response element (Nrf2/ARE), nuclear factor kappa-light-chain-enhancer of activated B cells (NF-κB), and phosphoinositide 3-kinase/protein kinase B (PI3K/Akt). Given these multifaceted properties, understanding the diverse neuroprotective mechanisms of PTAs may provide valuable insights into their potential role in AD management.

## 2. Pentacyclic Triterpene Acid Chemistry

**Figure 1:**
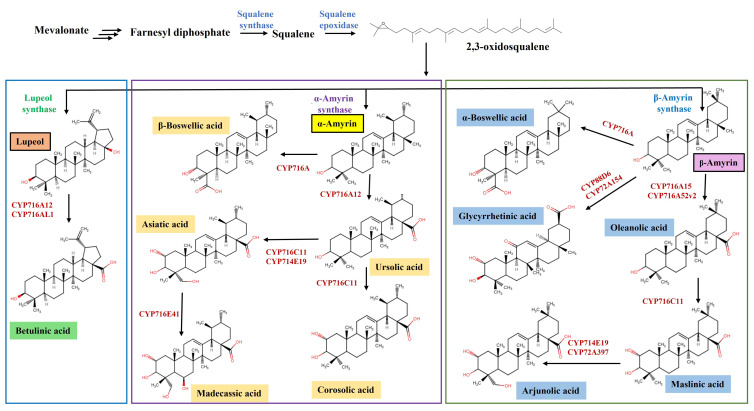
Biosynthetic pathway of several pentacyclic triterpeneic acids (PTAs). They are synthesized through the mevalonate pathway. Farnesyl diphosphate produces squalene, which was subsequently activated through epoxidation to produce 2,3-oxidosqualene. Various oxidosqualene cyclases (lupeol synthase and α/β amyrin synthase) convert 2,3-oxidosqualene through cyclization to produce numerous triterpene structures. Further oxidation by cytochrome P450s (CYP) subsequently leads to the formation of a particular PTA. Chemical structures were prepared using RCB PDB.

PTAs represented a subgroup of pentacyclic triterpenes with a carboxylic acid functional group and a 30-carbon backbone arranged into five fused alicyclic rings. Their biosynthesis started with the mevalonate route, resulting in the creation of farnesyl pyrophosphate (FPP). Two FPP molecules combine to generate squalene, which was subsequently activated through epoxidation to produce 2,3-oxidosqualene. Oxidosqualene cyclases (OSCs) convert 2,3-oxidosqualene through cyclization to produce numerous triterpene structures [14]. Additional alterations, such as oxidation by cytochrome P_450s_ (CYP_450_), subsequently resulted in the formation of particular PTAs [15,16,17] (Figure 1).

The three primary structural frameworks, oleanane-type (oleanolic acid, boswellic acid, glycyrrhetinic acid, and maslinic acid), ursane-type (asiatic acid and ursolic acid), and lupane-type (betulinic acid), were characterized by the placement of methyl (-CH_3_) groups and ring stereochemistry. These compounds generally contain a carboxylic acid (-COOH) group at the C28 (or C30) position, which was essential for their categorization as triterpene acids and was often accompanied by polar modifications, such as hydroxyls at C2, C3, C11, or C24. The presence of the -COOH group together with other functionalities (hydroxyl, keto, or double bond) modulateds the amphiphilicity, solubility, membrane permeability, and reactivity. The central pentacyclic structure provideds rigidity and metabolic stability, whereas modifications on the periphery determined several biological activities, including antioxidant, anti-inflammatory, and neuroprotective effects. For example, a hydroxy at C24 increaseds cyclooxygenase-2/5-lipoxygenase (COX-2/5-LOX) inhibitory activity, and a carbonyl at C11 createds synergistic effects with the acid moiety to further increase 5-LOX inhibition [18]. The modifications at C3 (hydroxyl), C17 (acid), or the C20 double bond improve TGR5 (G protein-coupled bile acid receptor) agonist activity [19]. Therefore, the pentacyclic structure in the TAs serveds as a suitable backbone, and polar alterations improve the bioactivity and pharmacokinetic features, making them favored scaffolds in drug development. The basic properties of some PTAs were presented in Table 1.

## 3. Mechanism of Neuroprotection in AD

### 3.1. Asiatic Acid

Asiatic acid (AA) was an ursane-type PTA (C_30_H_48_O_5_; MW 488.7 g/mol) obtained from *Centella asiatica* (Gotu kola/Indian pennywort) [20]. Structurally, it had three hydroxyl groups (C2, C3, and C23) and a carboxylic acid (C28) function that contributed to its therapeutic properties. It was patented (EP 0383171A2; WO 1998/023278A1) as a treatment for dementia and an enhancer of cognition in the year 1999 [21]. AA exerted its effects via various mechanisms, including antioxidant properties, anti-inflammatory actions, protection of mitochondria, and prevention of neuronal apoptosis [22,23]. The essential prerequisite for a compound to show a neuroprotective effect depended on its ability to cross the BBB. The in vitro apparent BBB permeability of AA (Papp) was reported as 50.94 ± 10.91 × 10^−6^ cm/s in primary porcine brain endothelial cells (PBECs), indicating efficient crossing of the BBB. In addition, it was cytoprotective against oxidative stress and passed the BBB without damaging the PBEC’s tight junction [24]. AA’s lipophilic backbone (*p*-value 4.0–5.5) and small size were better suitable for passive diffusion across the BBB than its glycosylated derivatives, as aglycones usually exhibited better permeability through the biological membrane than polar saponins [24,25]. Studies suggested that AA acted as a substrate of the major BBB efflux transporter, P-gp [26], which actively pumped AA out of the brain and into the bloodstream, thus inhibiting AA accumulations in CNS. As unmodified AA was susceptible to rapid P-gp-mediated efflux, specialized drug delivery systems were being explored to improve BBB retention and to enhance CNS uptake [26].

In AD, there was a marked rise in voltage-dependent anion channel (VDAC1) levels in the brain, which was related to mitochondrial dysfunction, oxidative stress, and synaptic deterioration [27]. Pretreatment with AA (0.01–100 nM) protected human neuroblastoma SH-SY5Y cells against toxicity by downregulating the expression of VDAC [28,29], thus protecting healthy mitochondrial function [30]. In a related study, AA (1 μM) in the neuroblastoma B103 cell line protected against amyloid-beta (Aβ_25–35_)-induced cell death [31]. Inhibiting the Aβ–VDAC1 interaction or lowering VDAC1 levels reduced Aβ-induced toxicity, emphasizing VDAC1 as a possible therapeutic target in AD [32]. AA also protected against rotenone- and α-synuclein-induced neurotoxicity in both in vitro and in vivo models by preventing mitochondrial permeability transition pore (MPTP) opening and inhibiting α-synuclein translocation to mitochondria [22].

Ceramides, produced from sphingosine, were involved in triggering the death of nerve cells in neurodegenerative diseases (NDs), like AD [33]. In rat brain cells, AA (0.01 and 1.0 μmol/L) reduced cell death and helped maintain mitochondrial membrane potentials (MMP) in a dose-dependent way. It also lowered the produced ROS after C2-ceramide exposure. At the highest dose (1.0 μmol/L), AA partially blocked apoptotic pathways by reducing the release of an apoptotic-regulating serine protease, high-temperature requirement protein A2 (HtrA2/Omi), into the cytosol [34]. AA also reduced the levels of Bax (a pro-apoptotic protein) and caspase-3 (a key executioner of caspases in apoptosis) and prevented the deactivation of extracellular signal-regulated kinase 1 and 2 (ERK1/2, a survival signaling pathway) [34]. In addition, AA reduced oxidant-induced neuronal apoptosis by modulating the protein kinase B (PKB)/GSK-3β (glycogen synthase kinase-3 beta)/caspase-3 signaling pathway [35] Specifically, AA promoted GSK-3β phosphorylation, which suppressed caspase-3 activation and enhanced neuronal survival.

Aluminum (Al) was viewed as a contributing factor to several neurological NDs, including AD and PD. Its neurotoxic effects can lead to oxidative damage in the brain, trigger apoptosis, and ultimately result in irreversible damage to neurons [36]. Studies suggested that AA administration reduced AlCl_3_-induced cell death by diminishing mitochondrial dysfunction, oxidative stress, and inflammation in both cellular and animal models of AD [37,38].

The cAMP response element binding protein (CREB) and its phosphorylated variant (pCREB) were crucial in improving dendritic arborization and modulating genes related to various neuronal functions, including synaptic plasticity, to boost memory and enhance cognition [39]. Reduced levels of pCREB were reported in AD, and were linked to cognitive decline, and serve as a potential AD biomarker [40]. AA (10 μg/mL) co-treatment boosted CREB phosphorylation over 2-fold in primary cortical cells and sustained increased pCREB levels through the activation of the mitogen-activated protein kinase/extracellular signal-regulated kinase/ribosomal S6 kinase (MAPK/ERK/RSK) pathway [41].

The human evidence was early and indirect rather than definitive for AA’s role in AD. In one phase 1 PK/PD study, a standardized *Centella asiatica* aqueous extract (CAP) was given as a single 2 g or 4 g dose to four mildly demented older adults already taking cholinesterase inhibitors; it was generally well tolerated. AA and madecassic acid were detected in plasma, and NRF2-related gene expression changed in a time course that matched the pharmacokinetic signal [42]. A separate ongoing phase I randomized trial was testing a *Centella asiatica* preparation in 48 adults (60–85 years) with mild cognitive impairment (MCI) or mild AD over 6 weeks, mainly to assess safety, tolerability, and targeted engagement, such as effects on neuronal viability, oxidative stress, and mitochondrial function [43]. In addition, a meta-analysis of 11 randomized trials did not find clear improvement across cognitive domains, but mood-related outcomes were better with the treatment [44].

### 3.2. Betulinic Acid

Betulinic acid (BTA) was a lupeol-type TPA (C_30_H_48_O_3_; MW 456.7 g/mol) and was a key component in various plants, including *Bacopa monnieri* (Brahmi/water hyssop) and *Betula alba* (white birch bark) [45]. BTA was used in the Indian traditional medicine system (Ayurveda) for treating CNS conditions [46] and to treat intestinal disorders, skin infections, and colds [47]. It had important pharmacological properties, such as antiviral, immunomodulatory, antioxidant, anti-inflammatory, and anticancer properties [48,49]. BTA had a distinctive exocyclic double bond (C20=C29), a hydroxyl at C3, and a carboxylic acid at C28. These functional groups offer sites for structural alterations that enhanced the drug’s pharmacological activity, targeted specificity, and increased solubility. Its adaptable scaffold was still a useful lead molecule in medication development, especially in the fields of neurology and oncology.

BTA had a high brain-to-blood partitioning ratio (C_brain_/C_blood_) of 8.20, indicating that it can easily cross the BBB [50]. Studies reported that even drugs bound to proteins could cross the BBB [51]. Since BTA was fully bound to proteins in blood, it likely entered s the brain either by detaching from the protein, staying attached, or both [50]. Its ability to cross the BBB made it a suitable candidate to treat neurological pathologies. Evidence also indicateds that BTA was not a P-gp substrate, and it may interact and inhibit ATP-binding cassette (ABC) transporters, including P-gp and BCRP, which could further influence its CNS availability [52,53].

The buildup of amyloid plaques in the brain was considered a major cause of dementia, leading to serious memory loss and symptoms like anxiety, depression, and poor motor coordination. Many studies showed that the soluble forms of Aβ cause more harm to brain cells in AD than the larger, insoluble plaques [54,55]. Aβ started as single units (monomers) and then clumps together to form insoluble fibrils through a process that depended on an initial trigger (nucleation). A study showed that BTA rapidly promoted the formation of Aβ42 fibrils while reducing the accumulations of toxic oligomeric intermediates, which were considered more neurotoxic than mature fibrillar forms [56]. While promoting fibril formation may reduce the accumulation of highly neurotoxic oligomeric intermediates, the long-term consequences of enhanced fibrillar deposition remain unclear. Even though mature fibrils were supposed to had relatively lower synaptotoxicity compared to soluble oligomers, increased plaque formation can still play a role in chronic neuroinflammation and stress on neurons. Therefore, this strategy should be considered carefully and needs to be validated more fully to assess the safety and clinical significance of this strategy.

The distinctive capacity of BTA to enhance fibrils, while reducing oligomers, combined with its established pharmacological characteristics positions BTA as a promising therapeutic option for AD. Pretreatment with BTA displayed a protective effect on behavioral impairments and hippocampal long-term potentiation (LTP) deficits in AD-induced rats with the strongest effect observed at a molar ratio of 1:4 (Aβ: BTA). In addition, it also restored the field excitatory postsynaptic potential (fEPSP) slope and population spike (PS) amplitude or area under the curve (AUC) in AD rats [57], indicating improvements in synaptic plasticity and neuronal communication. This effect may be due to reduced Aβ oligomer formation or the compaction of fibril plaques. Notably, BTA wasidentified as a potent activator of the proteasome, enhancing its chymotrypsin-like activity [58]. Since the proteasome also played a role in degrading intracellular Aβ, its activation was considered beneficial [59]. Based on this evidence, BTA may exert its therapeutic effects in dementia not only by reducing toxic Aβ oligomers but also by enhancing proteasome function and contributing to a reciprocal protective mechanism.

Molecular docking studies revealed that BTA strongly (−5.54 and −46.79 kcal mol^−1^) bound to Aβ and AChE, respectively [60], which could be used as a therapeutic option for the treatment of AD. In vitro, BTA inhibited both AChE (IC_50_ 24.2 ± 0.99 μM) and butyrylcholinesterase (BChE: IC_50_ 19.1 ± 1.33 μM), the two enzymes that break down acetylcholine (ACh) [61]. This dual action helped to increase ACh levels in the brain, which may improve memory in AD. BTA also demonstrated specific inhibitory effects on phosphodiesterases (PDEs) in vitro [62]. This inhibition restored levels of cyclic AMP (cAMP) and cyclic GMP (cGMP) in the hippocampus, which might support better cognitive function. Since PDE inhibition played a role in enhancing memory and learning, it was considered a promising therapeutic strategy for AD [63].

Mitochondrial dysfunction and the overproduction of ROS were strongly implicated in the pathogenesis of AD. It can trigger neuroinflammation, a process that could further damage neurons and exacerbate AD pathology [64]. Excessive ROS production was common in both Aβ- and streptozotocin (STZ)-induced animal models of AD [57,65]. In AD rats, microvascular function and BBB integrity were significantly impaired by STZ induction but were improved by BTA pretreatment. In addition, it also increased brain-derived neurotrophic factor (BDNF) expressions and reduced pro-inflammatory cytokine levels in the hippocampus [65]. In a related study, BTA was able to refurbish STZ-mediated behavioral, biochemical, and neurochemical alterations in rat brains by anti-inflammatory and antioxidant mechanisms [66]. Studies indicated that BTA offered some protection against brain [67] and retinal [68] damages caused by reduced blood flow and oxygen (ischemia-reperfusion injury) in mice, mainly by lowering oxidative and nitrosative stress. The protective effect of BTA pretreatment through mitochondrial c-Jun N-terminal kinase (JNK) and p38 mitogen-activated protein kinase (MAPK) signaling pathways was observed in mice splenocytes [69,70]. Furthermore, BTA significantly attenuated inflammation by shifting microglia from a pro-inflammatory M1 state to an anti-inflammatory M2 phenotype via a Ca^2+^/calmodulin-dependent protein kinase kinase-β/AMP-activated protein kinase (CaMKKβ/AMPK)-dependent pathway [71]. Though these studies were not conducted in AD models, it was very likely that BTA could also help reduce ROS and neuroinflammation in AD. But despite encouraging preclinical findings, clinical studies on BTA were still lacking.

### 3.3. Boswellic Acid

Boswellic acids (BAs) were oleanane/ursane-type PTAs (MW~512–528 g/mol) found in the gum resin (commonly known as frankincense) of Boswellia species, including *B. serrata* and *B. carterii* [68]. BAs were a structurally diverse group of triterpenoids differing mainly in acetyl and keto groups. These chemical differences dictated their biological activity, membrane permeability, and pharmacokinetics. In the ancient herbal system of medicine, BAs were used for treating colds, coughs, asthma, sores, wound healing, and arthritis [72]. Their pharmacological effects were diverse and included anti-inflammatory [73], antioxidant [74], antimicrobial [75], anticancer [76,77], and anti-excitotoxic [78] properties. These characteristics might have therapeutic implications for neurological conditions [79]. Their high lipophilicity made them promising candidates for targeting lipid-rich tissues like the brain, though poor solubility limits oral bioavailability.

All major BAs can cross the BBB to some degree with non-keto types (β-BA, α-BA) achieving significantly higher brain concentrations after oral dosing in rodents in comparison to the keto derivatives 11-keto-β-boswellic acid (KBA) and acetyl-11-keto-β-boswellic acid (AKBA) [80]. In preliminary rat studies, the brain-to-plasma ratios for KBA and AKBA were relatively low (~0.51 and 0.81), despite their lipophilic nature [81]. Their transport appeared to involve saturable carrier-mediated mechanisms rather than simple passive diffusion. The keto-BAs were found to inhibit Pgp activity in cell models [80,81]. Although KBA and AKBA could inhibit P-gp in vitro, their poor oral bioavailability and extensive metabolism result in low plasma concentrations, making significant BBB-related drug interactions clinically unlikely [80].

After oral administration of *B. serrata* extract (240 mg/kg) in rats, multiple BAs were detected (β-BA~1066 ng/g, α-BA~485 ng/g, AKBA~37.5 ng/g, and KBA~11.6 ng/g) in brain tissue [80]. It demonstrated that these compounds were bioavailable to the CNS after oral administration. Permeability studies in Caco-2 cells showed that AKBA had poor absorption, while KBA showed moderate absorption with a P(app) value of 1.69 × 10^−6^ cm/s [82]. However, both compounds affected the activity of organic anion transporting polypeptide 1B3 (OATP1B3) and multidrug resistance-associated protein 2 (MRP2) transporters [82]. OATP1B3 was not a major BBB transporter, but its modulation can alter liver drug uptake and systemic availability. MRP2 transporters limit brain penetrations of various compounds by pumping them out of the brain. Their ability to modulate OATP1B3 and MRP2 suggesteds potential drug–drug interactions, especially with other anionic drugs (like statins and methotrexate). Their inhibitory effect on these transporters can influence plasma or brain levels of co-administered drugs.

AD was closely associated with oxidative stress and persistent neuroinflammation, both of which can cause progressive neurodegenerations. Nrf2 and NF-κB were two transcription factors that regulate these processes [83]. Nrf2 was the master regulator of antioxidant response and boosts protective genes (heme oxygenase-1: HO-1; NAD(P)H quinone oxidoreductase 1: NQO1), reducing ROS and inflammation. NF-κB, in contrast, drives inflammation by producing cytokines (TNF-α, IL-1β, and IL-6), aggravating neuroinflammation, synaptic damages, and neuronal loss in AD [84]. AKBA, a bioactive compound from *Boswellia serrata*, demonstrated its neuroprotective effects in AD models [85,86,87]. It exerted its effects by modulating both Nrf2 and NF-κB pathways [87]. It upregulated Nrf2 and HO-1 expressions, enhancing the antioxidant defense system. Simultaneously, AKBA inhibited the phosphorylation of the inhibitor of NF-κB (IκBα) and the NF-κB p65 subunit, thereby suppressing the inflammatory response [87]. In lipopolysaccharide (LPS)-induced neuroinflammation models, AKBA markedly decreased key pro-inflammatory mediators (5-lipoxygenase: 5-LOX, TNFα, and IL-6) and increased IL-10 in rodent brains, related to improvedd cognition [88]. BAs revealed also potent anti-inflammatory effects by reversibly inhibiting (IC_50_ 3–10 µM) microsomal prostaglandin E2 synthase-1 (mPGES-1), the terminal enzyme in the biosynthesis of prostaglandin E2 (PGE2), reducing inflammation [89]. Moreover, when it was administered alongside cyclooxygenase (COX) inhibitors, it synergistically protected against oxidative-stress-induced neuronal damages and cognitive decline by reducing inflammations and decreasing glutamate levels [90]. AKBA improved levels of BDNF while decreasing glial fibrillary acidic protein (GFAP) in the hippocampus and promoting cognition [88]. BDNF playeds a central role in learning and memory, as its reduced levels were linked to cognitive deficits and AD progression [91]. On the other hand, GFAP was an astrocytic cytoskeletal protein, and its increased expressions were correlated with increased Aβ plaque density in AD brains [92]. BA also protected the cells from tau toxicity in cellular and in vivo studies [92,93]. Since protein phosphatase 2 (PP2A) was the major tau phosphatase that dephosphorylates tau and cyclin-dependent kinase 5 (CDK5) was a key kinase that hyperphosphorylates tau, the observed reduction in tau phosphorylation by BA suggesteds it may either enhance PP2A activity, inhibit CDK5 activity, or activate GSK-3β [94]. Supporting this possibility, betulinic acid hydroxamate (BAH) showed neuroprotective effects through a PP2A-dependent mechanism in the activation of the hypoxia-inducible factor-1α -1α (HIF-1α) pathway [95]. In addition, AKBA attenuated tau oligomer-induced cytotoxicity and improved cell viability, further supporting the therapeutic potential of BA compounds in reducing tau toxicity pathways.

In animal studies, oral administration of 0.5% BA in an AD rat model ameliorated hippocampal antioxidant enzymes (superoxide dismutase: SOD, catalase: CAT, glutathione peroxidase: GPx), reduced glucose transporter 2 (GLUT2) expression, and lowered hippocampal degeneration in BA-treated groups compared to the untreated group [96]. GLUT2, mostly present in astrocytes, exhibited substantial upregulations in the hippocampus of AD brains. Astrocyte activation, a typical characteristic of AD, was probably the cause of this rise. In AD brains, GLUT2 expression was noticeably higher than GLUT1 and GLUT3 levels. This raiseds the possibility that GLUT2 playeds a part in AD progressions and might be connected to both neuronal dysfunction and poor glucose metabolism [97]. Decreased cholinergic neurotransmission caused the development of Aβ plaque and raised tau phosphorylations, eventually causing cortical dysfunction, memory loss, and task learning difficulty in AD [98,99]. Also, BA pre- and co-treatment improved levels of neurotransmitters ACh (1.7- and 1.5-fold) and dopamine (1.3- and 1.2-fold) in the rat hippocampus compared to the AD group, thereby improving cognition in the treated groups [96]. These results were supported by a previous study, where administration of BA (160 mg/kg, i.p.) in rats inhibited AChE activity and malondialdehyde (MDA) levels (a marker of lipid peroxidation) and increased glutathione (GSH) contents in the cerebral cortex [100], improving spatial learning and cognition in the treated group.

Clinical evidence for BAs remained limited, but a few human trials suggested that few *Boswellia*-derived preparations may have measurable effects on cognition or related outcomes. A double-blind, placebo-controlled clinical trial (*n* = 85, age < 65 years) with mild-to-moderate AD evaluated BA (K-Vie™, 3 × 400 mg/day/oral) for 6 months [101,102]. The treatment improved cognitive (clinical dementia rating-sum of boxes (CDR-SOB) and mini-mental state examination (MMSE)) and neuropsychiatric scores while reducing inflammatory cytokines and altering plasma AD biomarkers (Aβ42/Aβ40 ratio). However, the study was limited by its relatively small sample size, short duration, and lack of long-term follow-up, necessitating larger multicenter clinical studies for validation [101,102]. Two additional human studies, although not in AD, also suggested potential cognitive benefits of *Boswellia* extracts. In a double-blind, placebo-controlled trial (IRCT20170315033086N5), *Boswellia serrata* (K-Vie™) was tested for 3 months in patients (*n* = 46) recovering from traumatic brain injury (TBI) and was associated with improvements in memory, attention, and executive function measures [103]. Separately, a placebo-controlled study in multiple sclerosis (MS) patients (*n* = 80) with cognitive impairment (IRCT2013070813911N1) found that *Boswellia papyrifera* (300 mg/twice a day/2 months) improved visuospatial memory, while effects on other cognitive domains were limited [104]. Together, these findings indicate that Boswellia-derived preparations may have cognition-modulating effects beyond AD, but the available evidence remaineds preliminary and indication-specific.

### 3.4. Glycyrrhetinic Acid

Glycyrrhetinic acid (GA), an oleanane-type PTA [C30H46O4, MW 470.68 g/mol], was a hydrolytic product of glycyrrhizin, the major bioactive compound in licorice (*Glycyrrhiza glabra*) [105]. Structurally, it featured a five-ring backbone with a hydroxyl group at (C3) and a carboxylic acid (C30), existing primarily in the bioactive 18β-glycyrrhetinic acid (18 β-GA) form. GA exhibited diverse pharmacological properties, including anti-inflammatory [106], antidiabetic [107], hepatoprotective [108], anticancer [109], and neuroprotective [110] effects.

Glycyrrhizic acid was hydrolyzed by intestinal bacteria into GA, which was then absorbed and metabolized in the liver into glucuronide and sulfate conjugates. These metabolites were processed in the enterohepatic circulation, where they were excreted into bile, converted back to GA in the intestine, and reabsorbed, resulting in delayed plasma clearance [111,112]. GA was reported to inhibit P-gp and multidrug resistance-associated protein 1 (MRP1) activity, potentially improving the effectiveness of chemotherapy by reducing multidrug resistance [113,114]. After a 600 mg dose, GA could still be detected in urine for up to 2–4 days, indicating slow clearances and prolonged circulations [115]. Gastrointestinal transit time also influenced its reabsorption and may contribute to side effects such as hypertension [116]. GA also demonstrated moderate BBB permeability. After oral administration of licorice root in rats, GA was detected in the brain, plasma, and cerebrospinal fluid. In vitro BBB models further showed a permeability rate of 13.3% and Papp of 16.5 × 10^−6^ cm/s, supporting its CNS penetration [117]. Increased BBB permeability under ischemic conditions may further enhance its neuroprotective effects through modulation of autophagy and glutamate transport [118].

In the context of AD, GA demonstrated multifaceted neuroprotective actions. Aging, toxic tau oligomers (TauO), and long-term brain inflammation were key contributors to AD [119]. TauO triggereds this by releasing high mobility group box 1 (HMGB1), which in turn activateds an inflammatory response (senescence-associated secretory phenotype: SASP) [120]. GA blockeds HMGB1 release by inhibiting the p38 MAPK and NF-κB pathways, both crucial for SASP [120]. In aged tauopathy mice (12 months old), combined ethyl pyruvate (EP) and GA treatment reduced TauO buildup, senescent cells, and brain inflammation, ultimately improving memory [120]. GA and 18β-GA inhibited BACE1 (IC_50_ 20.12 ± 1.87 µM and 8.93 ± 0.69 µM, respectively), thereby reducing Aβ production, a central feature of AD pathology [121].

18α-GA exerted its neuroprotection against proteotoxic stress in a *C. elegans* model of AD and SH-SY5Y cells treated with the Aβ peptide [122] by enhancing proteasomal degradation of Aβ species. Likewise, *Glycyrrhiza uralensis* water extract (GWE) improved cognitive impairment in Aβ_25–35_-induced mice by reducing ROS levels in the brain [123]. To increase effectiveness, Gad et al. [124] suggested the use of lipid nanocapsules for intranasal delivery of GA, as it improved cognition and behavior in scopolamine-treated animal models by reducing oxidative stress (CAT and SOD). In a rat model of vascular dementia from induced bilateral common carotid artery occlusion, glycyrrhizic acid (20 mg/kg for 5 days) improved spatial memory and long-term potentiation while reducing oxidative stress markers in the hippocampus and cortex. It also increased SOD activity and inhibited voltage-gated sodium channels [125]. In aged mice (16–18 months), treatment with glycyrrhizic acid (25–50 mg/kg for 8 weeks) also improved cognitive performance, increased synaptic proteins such as PSD95 and synaptophysin, and modulated cholinergic signaling [126]. A review by Zeng et al. further stressed the neuroprotective effects of GA across rodent models of AD, Parkinson’s disease, and Huntington’s disease, particularly through reducing neuroinflammation and oxidative stress [110].

### 3.5. Maslinic Acid

Maslinic acid (MA) [C_30_H_48_O_4_, MW 472.7 g/mol], also known as (2α,3β)-2,3-dihydroxyolean-12-en-28-oic acid, was an oleanane-type pentacyclic triterpenoid primarily found in the skin of olives (*Olea europaea*) and various other plants [127]. Structurally, it had hydroxyl groups at the 2 and 3 positions, a carboxylic acid at C28, and a double bond between C12 and C13, classifying it as an oleanane-type triterpene. MA was a promising nutraceutical with several biological activities, including anti-inflammatory, antioxidant, anti-parasitic, anti-tumor, cardioprotective, and hypoglycemic [128,129]. An ADMET-based model predicted low BBB penetration (log BB −0.493) and moderately low CNS permeability (log PS −1.477) for MA, indicating limited diffusion through the BBB [130]. However, the neuroprotection displayed by MA in preclinical models of cerebral ischemia [131,132] suggested that it had minimal BBB penetration or could exert CNS effects indirectly through peripheral anti-inflammatory and antioxidant actions. Yet, the computational models often failed to account for factors like active transport, protein binding, species differences, and disease-related BBB alterations that could strongly influence brain penetration in vivo [133,134]. As a result, the predicted low BBB permeability may not always match the neuroprotective effects observed in experimental models, including ischemia studies [135]. Therefore, experimental validation remaineds essential to accurately assess CNS availability and therapeutic potentials.

Aβ-induced activation of NADPH oxidase (NOX), followed by increased oxidative stress, was observed in AD. This pathway can disrupt cellular function, damage neuronal structures, and contribute to neuronal cell death, a hallmark of AD. Aβ-induced NOX activation in microglia can lead to inflammation and neuronal damage [136]. Pretreatment with MA (2–16 µM) significantly lowered the expressions of NOX subunits (gp91phox, p47phox), causing lower ROS levels and reduced levels of inflammatory cytokines (TNF-α, IL-1β, and IL-6) [137] in nerve growth factor-differentiated PC12 neuronal cells. Aβ upregulated the receptor for advanced glycation end products (RAGE), which contributed to AD pathology by activating downstream MAPK and NF-κB pathways, thereby enhancing ROS and cytokine production [138]. MA pretreatment attenuated Aβ-induced RAGE expressions, which were associated with decreased activation of NF-κB, p38 MAPK, and ERK1/2 signaling. This cascade ultimately caused a reduction in ROS production and inflammatory cytokine release [137]. MA exerted anti-inflammatory effects in cultured cortical astrocytes by suppressing NF-κB, COX-2, and inducible nitric oxide synthase (iNOS) at protein and mRNA levels [139].

In AD, disrupted synaptic connectivity and axonal degeneration strongly correlate with memory impairment and cognitive decline [140]. MA promotedd axonal regeneration and increased synaptophysin (synaptic marker) by activating the PI3K/Akt pathway and inhibiting GSK-3β in a brain ischemia model [132,141]. Dysregulation of GSK-3β in AD causeds tau hyperphosphorylation and synaptic breakdown, two important characteristics of AD pathogenesis; hence, inhibiting this protein was essential [140]. Since GSK-3β was a major tau kinase involved in tau pathology, MA-mediated modulation of this pathway may also help preserve microtubule stability and reduce tau-associated neuronal dysfunction. MA might help re-establish neural connections and prevent tau-associated neurodegeneration, providing a multitarget strategy for AD therapy. In addition, MA demonstrated neuroprotective potentials by indirectly mitigating glutamate-induced toxicity in primary cultures of cortical neurons. MA enhanced the glutamate clearance capacity of astrocytes by regulating the expressions of glutamate aspartate transporter (GLAST) and glutamate transporter-1 (GLT-1) [142]. This effect reduced extracellular glutamate concentrations, thereby protecting neurons from excitotoxic damage in co-culture models. Since excitotoxicity contributeds to synaptic dysfunction and neuronal death in AD [143], the ability of MA to improve astrocytic glutamate uptake suggesteds a promising mechanism by which it may attenuate neurodegeneration and cognitive decline in AD.

Scopolamine-induced cognitive decline in animals served as a pharmacological model mimicking the molecular alterations and cognitive impairments seen in AD [144]. MA reversed scopolamine-induced memory deficits in mice by binding to the tyrosine protein kinase B (TrkB) receptor, thereby activating BDNF and downstream pathways (ERK/CREB) in the hippocampus to enhance cognition [145]. In addition, the neurotransmitter ACh played a crucial role in learning and memory, and its concentration was markedly reduced in AD [128]. Although native MA showed limited cholinesterase inhibition, its derivatives inhibited butyrylcholinesterase (BChE) and AChE in the low micromolar range [146], like galantamine, emphasizing MA’s potential as a base scaffold for medications that target the cholinergic system.

Briefly, MA exhibited neuroprotective effects in AD through various mechanisms by reducing inflammation, oxidative stress, and tau pathology and improving synaptic transmission and cognition in preclinical trials, but there were no published human trials.

### 3.6. Oleanolic Acid

Oleanolic acid (OA) was an oleanane-type pentacyclic triterpenoid [C_30_H_48_O_3_, MW 456.7 g/mol], also known as (2α,3β)-2,3-dihydroxyolean-12-en-28-oic acid [147]. Its pentacyclic ring had a hydroxyl group at C3, a double bond between C12 and C13, and a carboxylic acid group at C28. OA was abundant in olive leaves, apples, and various Ligustrum species (Forsythia) [140]. It had a wide range of biological properties, including neuroprotective [148,149], antimicrobial [150], antidiabetic [151], anticancer [152], wound healing [153], and anti-inflammatory [154] effects.

Permeability studies conducted using Caco-2 cells calculated the Papp value of 1.1–1.3 × 10^−6^ cm/s OA, suggesting poor absorption and transport through passive diffusion [155]. In addition, its interaction with P-gp may further reduced the absorption and tissue penetrations [156]. Despite poor transport in Caco-2, OA was reported to traverse the BBB and displayed neuroprotective and barrier-stabilizing effects, mostly through anti-inflammatory and junction-preserving effects by targeting p38MAPK/vascular endothelial growth factor (VEGF)/SRC proto-oncogene (Src) [157] and antioxidant [149] mechanisms. In subarachnoid hemorrhage models, OA maintained junctional protein expression by modifying high mobility group box 1/toll-like receptor 4 (HMGB1/TLR4) signaling through sirtuin (SIRT1) activation, improving barrier integrity [158]. These findings suggest that high BBB penetration was not required by OA to show neuroprotection. Its action on the endothelium or entry at low, but sufficient concentrations, can modulate signaling pathways.

#### 3.6.1. Antioxidant and Anti-Amyloidogenic Effects

OA acted as a strong antioxidant by removing harmful free radicals that damaged the cells. In vitro, it also reduced the Aβ buildup and inhibited AChE and BACE1, the key enzymes in AD [159]. In silico studies further showed strong binding of OA to the active site of AChE through CH-π interactions with aromatic residues, resulting in significant AChE inhibition (IC_50_~9 μM) [160]. In N2a/APP695swe cells, OA (10–25 μM) reduced ROS production, caspase-3 activity, Aβ levels, and cell death. At 10 μM, OA increased stanniocalcin-1 (STC-1) and uncoupling protein-2 (UCP2) expression, suggesting neuroprotection through regulation of oxidative stress and mitochondrial function by the STC-1/UCP2 pathway [161]. Both of these were protective proteins: STC-1 had anti-apoptotic, anti-inflammatory, and antioxidant roles [162,163], while UCP2 regulateds mitochondrial membrane potentials [164]. In a recent study, OA was reported to exert multitargeted effects in SH-SY5Y neuroblastoma cells overexpressing APP by reducing APP levels and oxidative stress through activation of the Nrf2/HO-1 pathway [152].

#### 3.6.2. Autophagy, Ferroptosis, and ER Stress Regulation

OA promoted autophagy by suppressing phosphorylated mammalian target of rapamycin (mTOR) and elevating markers like autophagy protein 5 (ATG5) and LC3-phosphatidylethanolamine conjugate (LC3-II) in APP-overexpressing SH-SY5Y cells [165]. Autophagy-enhancing effects may also support the clearance of aggregated tau species and limit intracellular tau accumulation. In addition, OA helped to restore levels of ferroptosis-related proteins (glutathione peroxidase 4: GPX4; nuclear receptor coactivator: NCOA; cyclooxygenase-2: COX2) and ER stress markers (glucose-regulated protein 78: GRP78; C/EBP homologous protein: CHOP), along with key ER stress pathways (inositol-requiring enzyme/X-box binding protein 1: IRE1/XBP1s; protein kinase R (PKR)-like endoplasmic reticulum kinase/eukaryotic translation initiation factor 2 subunit 1: PERK/eIF2α; and activating transcription factor 6: ATF6). OA further protected mitochondria by regulating proteins involved in mitochondrial dynamics (mitofusin 1 and 2: MFN1-2; OPA1 mitochondrial dynamin-like GTPase: OPA1; mitochondrial fission 1 protein: FIS1; and dynamin-related protein 1: DRP1). It also increased growth differentiation factor 11 (GDF11) expressions and reduced phosphorylations of human epidermal growth factor receptor (4ErbB4) and TrkB without altering BDNF levels [166].

#### 3.6.3. Anti-Inflammatory Effects

OA reduced neuroinflammation in LPS-induced BV2 microglia cells by reducing the release of pro-inflammatory mediators, such as IL-1β, IL-6, TNF-α, and nitric oxide (NO) and decreasing ROS. These effects were correlating to the downregulation of cytokine and iNOS gene expressions, along with the restoration of glutathione levels. It was suggested that OA exerted its anti-inflammatory and antioxidant effects by modulating the transcription factors Nrf2 and NF-κB, thereby enhancing the cell’s adaptive response to oxidative stress and inflammation [167]. OA also suppressed the inflammation, secretory phospholipase A_2_-IIA (sPLA2-IIA) expressions, and abnormal calcium influx in Aβ-activated astrocytes and ameliorated cognitive deficits in the rat model of Aβ-induced AD [168].

#### 3.6.4. Synaptic Plasticity and Cognitive Function

OA enhanced the phosphorylation of ERK1/2 and CREB and increased BDNF expressions in hippocampal neurons [169]. Since the ERK/CREB/BDNF signaling pathway was involved in synaptic plasticity and cognitive function closely, OA improved learning and memory in scopolamine-induced memory impairment in a mouse model by modulating this pathway through TrkB activation [165].

In short, the multitarget profile of OA, combining antioxidants, anti-inflammatory, mitochondrial protection, and AChE inhibition, madekes it a compelling lead for new AD therapies. However, no clinical trial was yet conducted to validate its efficacy and safety in patients with AD.

### 3.7. Ursolic Acid

Ursolic acid (UA) was a naturally occurring pentacyclic oleanane-type triterpenoid (C_30_H_48_O_3_, MW 456.7 g/mol). UA was found in the waxy coatings of many fruits, herbs, and medicinal plants, including *Rosmarinus officinalis*, *Ocimum sanctum*, and apple peels [170]. It structurally resembleds OA, differing only by the position of a methyl group at C19. UA had a hydrophobic pentacyclic ring system with a hydroxyl group (C3), a double bond (between C12 and C13), and a carboxylic acid group (C28) [171]. This configuration contributeds to its poor water solubility but high lipophilicity, influencing its bioavailability and interactions with lipid membranes. UA displayed anti-tumor [172,173], anti-inflammatory [174], antioxidant [175], antidiabetic [176], antimicrobial [175], neuroprotective [177,178], and hepatoprotective [179] effects in several studies.

Due to its lipophilic pentacyclic triterpenoid structure, UA can diffuse across lipid membranes and passively enter the brain. Pharmacokinetic studies in rodents showed that, after oral dosing, UA rapidly appeared in plasma and was detectable in multiple organs, including the brain, suggesting effective BBB penetration [180]. Studies using Caco-2 and MDCK cell models suggested that UA was a substrate of P-gp and may also weakly inhibit P-gp at higher concentrations [181]. It additionally interacted with BCRP, influencing the pharmacokinetics of certain BCRP substrates [182]. Along with CYP3A4-mediated metabolism, these efflux mechanisms contributed to extensive intestinal clearance and poor systemic bioavailability of UA [181,183].

Aβ deposition and inadequate clearance in the brain accelerate AD progression. The proteasome assembly in eukaryotes was responsible for breaking down damaged and misfolded proteins to maintain proteostasis [184]. UA prevented Aβ-induced proteotoxic stress by reducing Aβ and increasing proteasome activity in Aβ-induced paralysis in *Caenorhabditis elegans* [185]. UA reduced Aβ accumulations by inhibiting BACE1 activity in vitro and downregulated amyloidogenic pathways, which were central to AD pathology [186]. Increasing evidence demonstrateds that oxidative stress and inflammation were involved in Aβ-induced memory impairments. In Aβ_25–35_-exposed PC12 neuronal cells, UA significantly protected against neuroinflammation and cell damage. It reduced the expressions of inflammatory enzymes (iNOS and COX-2) and blocked activation of the NF-κB pathway by preventing nuclear translocation of its p65 subunit and phosphorylation of IκB-α. UA enhanced autophagic clearance by increasing the phosphorylation of JNK and ameliorated motor and non-motor symptoms in a PD mouse model [187]. UA also inhibited stress-activated kinases ERK1/2, p38, and JNK, which contribute to inflammatory signaling [188]. It also prevented apoptosis, reduced oxidative stress, and inhibited caspase-3 in the same model [189]. It also blocked Aβ binding to the microglial CD36 receptor and reduced the productions of pro-inflammatory factors and ROS [190]. In addition, UA attenuated the inflammatory response induced by Aβ_25–35_ in vitro through the NF-κB signaling pathway [188]. UA drastically improved cognitive function and hippocampal health in Aβ-induced mice by reducing amyloid burden and restoring neurogenesis. Molecular docking further revealed that UA bound to neurogenesis-related proteins Ki-67 (a proliferating cell marker) and doublecortin (DCX; an immature progenitor cell marker) with binding energies comparable to donepezil [191]. In a similar study, UA significantly reversed the Aβ_25–35_-induced learning and memory deficits in mice by reducing inflammation (IL-1β, IL-6, and TNF-α levels) and oxidative stress (oxidized glutathione: GSSG and lipid peroxidation) in the hippocampus [160]. UA reduced tau hyperphosphorylation by suppressing the inflammatory complement component 3/complement component 3a receptor/GSK3β β (C3/C3aR/GSK3β) pathway without altering amyloid pathology, further supporting the role of PTAs in modulating key tau-regulating signaling mechanisms [192]. UA also improved cognition in AlCl_3_-induced rats by reducing the expression of inflammatory genes, inhibiting AChE, and improving levels of antioxidant enzymes (CAT and SOD) in the brain [193]. UA, as the main bioactive phytochemical of *P. incarnata* ethanolic extract, improved spatial memory performance and ameliorated Aβ_25–35_ accumulations by reducing microglial inflammations (ionized calcium-binding adapter molecule 1: Iba) in the mouse hippocampus [194].

In brief, UA improved cognitive performance and reduced Aβ plaque burden, oxidative stress, and inflammation in several in vitro and in vivo studies, making it a promising candidate for AD therapy. However, clinical trials evaluating its efficacy and safety were still lacking.

### 3.8. Miscellaneous

Several additional PTAs reveal their known neuroprotective properties, but the mechanisms of neuroprotection were not well established in AD-specific models. Further research would be necessary to characterize these PTAs in AD contexts.

Arjunolic acid (ARA) [C_30_H_48_O_5_, MW 488.7 g/mol] was an ursane-type pentacyclic triterpenoidal saponin primarily isolated from *Terminalia arjuna*, a plant widely used for its cardioprotective properties in Indian traditional medicine [195]. The literature suggesteds its neuroprotective role, but no study was available on AD. ARA provided neuroprotection in cerebral ischemia/reperfusion injury (I/R) in animal models by regulating oxidative stress markers (MDA, NO, GSSG) and enzymes (CAT and SOD) [196]. It also suppressed neuroinflammation through modulating the SIRT1/AMPK/Notch1 signaling pathway in an LPS-induced mouse model. In the same study, BDNF and serotonin were significantly increased in the mouse hippocampus [197]. Even though these studies did not specifically focus on AD, the neuroprotective mechanism displayed by ARA could be designed in future AD studies.

Corosolic acid (CA) [C_30_H_48_O_4_, MW 472.7 g/mol] was an ursane-type pentacyclic triterpenoid derivative of UA, commonly found in various plants, notably *Lagerstroemia speciosa* (Banaba) and rosemary. It was also referred to as “phyto-insulin” due to its glucose-lowering properties [198]. The use of CA in AD was supported by preliminary analysis on a computational basis, as it can bind to the tau protein to prevent the fibrillar network [199]. A stable form of CA, di-acetylated CA (diAcCA), reduced inflammation and oxidative stress, increased neuronal synaptic density, and decreased phosphorylated-tau aggregates and Aβ plaques in 5xFAD animal models of AD [200].

Madecassic acid (MAA) [C_30_H_48_O_6_, MW 504.7 g/mol] was a lupane-type pentacyclic triterpenoid. It was a bioactive component of *Centella asiatica*, a plant that was used in Eastern medicine to enhance cognitive performance [201]. MAA effectively inhibiteds AChE (IC_50_ 17.83 ± 0.06 µg/mL) and interacteds with the active site of the enzyme [202]. In preclinical research, water extracts from *C. asiatica* (CAW) enhanced cognition in 5xFAD mouse models of AD and aging by modifying mitochondrial biogenesis and triggering genes involved in the Nrf2-dependent antioxidant response. AD pathogenesis was linked to Nrf2’s regulation of antioxidant response genes and modulation of mitochondrial function. Continued treatment with CAW activated the Nrf2-regulated antioxidant response pathway, decreased Aβ pathology, and improved memory [203]. MAA displayed anti-inflammatory and antioxidant activities [204,205], which were relevant to mechanisms in AD; nevertheless, direct studies specifically on MAA’s role in AD were limited.

Table 2 summarized these findings with their effects in consistent experimental evidence.

**Figure 2:**
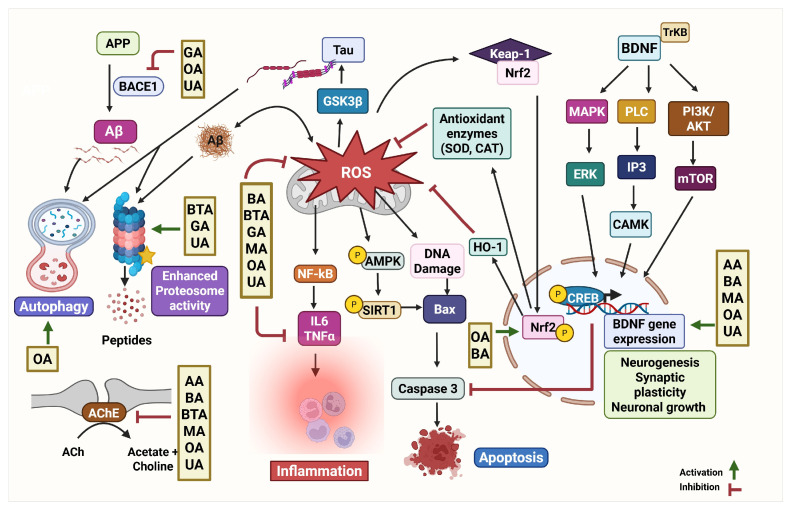
A comprehensive overview of the multimodal neuroprotective effects of PTAs in AD. BACE1 (β-secretase 1) and AChE (acetylcholinesterase) were both implicated in AD and were considered promising drug targets. BACE1 was involved in the production of amyloid-β (Aβ) peptides from amyloid precursor protein (APP). Aβ was a key component of amyloid plaques found in the brains of AD patients. AChE, on the other hand, breaks down acetylcholine (ACh), a neurotransmitter important for learning and memory. Mitochondria, the powerhouses of cells, were vulnerable to stress. In AD, this damage causeds the overproduction of reactive oxygen species (ROS). In turn, ROS causes oxidative stress, damaging cellular components like proteins, lipids, and DNA. The interplay between mitochondrial ROS, nuclear factor kappa-light-chain-enhancer of activated B cells (NF-κB) activation, and production of pro-inflammatory cytokines (tumor necrosis factor alpha: TNF α; and interleukin 6: IL6) resulteds in neuroinflammation, DNA damage, and apoptosis, which create a vicious cycle in AD. ROS can also activate several key signaling pathways, including AMP-activated protein kinase/sirtuin 1 (AMPK/SIRT1), and the apoptotic machinery (Bcl2-associated X: Bax and caspases). ROS can activate glycogen synthase kinase 3 beta (GSK3β), which in turn can cause tau phosphorylation. This process was implicated in the pathology of AD and other tauopathies. Activation of nuclear factor (erythroid-2 related) factor 2 (Nrf2) antioxidant signaling was frequently linked to ROS production through a process involving Kelch ECH-associated protein 1 (KEAP1). Translocation to the nucleus and binding of Nrf2 to the antioxidant response element (ARE) resulted in the production of heme oxygenase 1 (HO-1) and several antioxidant enzymes, including superoxide dismutase (SOD) and catalase (CAT), that inhibited ROS generation. The ubiquitin–proteasome system and the autophagy-lysosome pathways were dysfunctional in AD, as a result of tau and Aβ not being degraded and remaineding accumulated in the cells. AD and neurodegeneration were significantly influenced by the tropomyosin receptor kinase B/brain-derived neurotrophic factor/cyclic AMP response element-binding protein (TrkB/BDNF/CREB) pathway. By interacting with TrkB, BDNF starteds intracellular signaling pathways activating CREB that affect neuronal survival, plasticity, and cognitive function through the extracellular signal-regulated kinase/phosphoinositide 3-kinase/protein kinase B (ERK/PI3K/AKT) pathways or by increasing the production of neurotrophic factors, improving synaptic connections, and decreasing neurodegeneration. The pentacyclic triterpenic acids (PTAs) helped in modulating these enzymes/pathways at different steps, demonstrating a significant benefit in reducing Aβ, oxidative stress, neuroinflammation, and cell death and promoting autophagy and proteosomal degradation. Most importantly, they increase the production of neurotrophic factors and synaptic connections, hence improving cognition. [Red blunt-ended arrow: inhibition, green arrow: activation]. Abbreviation for PTAs: AA: Asiatic acid; BTA: Betulinic acid; BA: Boswellic acid; GA: Glycyrrhetinic acid; MA: Maslinic acid; OA: Oleanolic acid; UA: Ursolic acid. Created in Biorender.com.

Figure 2 summarizes the neuroprotective mechanism of action displayed by major PTAs in AD.

## 4. Translational Perspectives and Current Limitations

### 4.1. Multitarget Mechanisms of PTAs

As discussed, PTAs, including AA, BA, OA, GA, and MA, consistently targeted major AD-related pathological processes in cellular and animal models. These compounds reduced Aβ accumulations, limited tau hyperphosphorylations, and alleviated oxidative stress and neuroinflammations. Many studies also reported improvements in synaptic markers, such as BDNF, pCREB, and PSD95, along with better cognitive performance in behavioral tests, including the Morris water maze and novel object recognition tasks. In addition to Aβ pathology and neuroinflammation, AD progression was also associated with synaptic loss, mitochondrial dysfunction, impaired protein clearance, excitotoxicity, ferroptosis, and BBB damage. Several PTAs influenced kinase signaling pathways associated with tau pathology, including GSK-3β-related mechanisms and PP2A-associated neuroprotective signaling. The enhancement of autophagy and proteostasis may support the clearance of abnormal protein aggregates. Overall, PTAs showed protective effects against several interconnected pathways by reducing oxidative stress, regulating kinase signaling, improving autophagy, and supporting synaptic function, supporting their potential as multitarget agents for AD therapy. However, the mechanisms varied among PTAs. AA and UA showed stronger anti-amyloid effects, whereas BA, GA, and MA were more closely associated with anti-inflammatory and tau-related mechanisms through NF-κB, GSK-3β, PP2A-associated signaling, and CDK5-related pathways. These complementary actions suggest that combinations of PTAs may provide broader therapeutic benefits in AD.

### 4.2. Translational Comparison

Changes in biomarkers in PTA-treated AD models, including reduced IL-1β and TNF-α levels, enhanced antioxidant enzymes, and improved Aβ42/40 balance, paralleled certain biomarker changes reported with anti-amyloid and anti-tau antibody therapies. Clinical findings with BA also demonstrated improvements in plasma Aβ42/40 ratio and inflammatory cytokines, supporting the translational potential of certain PTAs. PTAs may exert part of their effects through peripheral anti-inflammatory actions or endothelial protection, especially considering their relatively modest BBB penetration.

### 4.3. Biomarker Limitations

Despite encouraging findings, current evidence for PTAs remained largely preclinical. Most studies relied on plasma or tissue biomarkers without validation using CSF biomarkers or amyloid-PET imaging in humans. In addition, many rodent studies used acute treatment paradigms that may not fully reflect chronic age-related AD pathology. Dose-response relationships and clinically achievable brain concentrations also remained poorly defined, particularly for compounds such as OA. Several PTAs showed beneficial effects at micromolar concentrations in vitro despite poor solubility, low oral bioavailability, rapid metabolism, efflux-mediated clearance, and limited BBB penetration. Therefore, long-term clinical studies integrating advanced biomarkers, such as p-tau217, GFAP, and NfL, would be needed to determine whether PTA-induced biomarker changes could truly predict slowing of AD progression.

## 5. Conclusions and Future Directions

AD remained a complex neurodegenerative disorder with limited disease-modifying therapies. This review summarized the neuroprotective effects of PTAs, including AA, BTA, BA, GA, MA, OA, and UA, which demonstrated antioxidant, anti-inflammatory, anti-amyloidogenic, mitochondrial-protective, and synaptic regulatory properties in cellular and animal models of AD. Several PTAs also improved learning and memory in behavioral studies, highlighting their multitarget therapeutic potential. However, most evidence remains preclinical, and the reported benefits were largely derived from in vitro systems or rodent models that may not fully represent the complexity of human AD. Clinical evidence is still limited to a few small-scale studies with short follow-up periods and insufficient validation using established AD biomarkers or neuroimaging approaches.

In addition, poor solubility, limited oral bioavailability, uncertain BBB penetrations, and incomplete pharmacokinetic characterization continue to restrict the translational applicability of many PTAs. Several compounds, including CA, MAA, and ARA, also lacked direct evaluations in AD-specific models, despite showing neuroprotective effects in other neurological conditions. Future research should, therefore, focus on rigorous mechanistic studies, standardized dose-response evaluations, long-term safety assessments, and well-designed clinical trials. Improving delivery strategies through nanoformulations, structural modification, or intranasal administration may further enhance therapeutic efficacy while reducing systemic limitations. Delivery systems, such as liposomes, polymeric nanoparticles, solid lipid nanoparticles, and nanoemulsions, were potential in improving the solubility, stability, and CNS delivery of several PTAs, particularly GA.

Overall, PTAs represented an interesting source of multitarget neuroprotective compounds, but substantial experimental and clinical validation was still required before their utility in AD therapy can be established.

## Figures and Tables

**Table 1:** Basic physicochemical properties of some pentacyclic triterpenic acids.

Property	Asiatic Acid	Betulinic Acid	Boswellic Acid (AKBA)	Glycyrrhetinic Acid	Maslinic Acid	Oleanolic Acid	Ursolic Acid
Skeleton Type	Ursane	Lupane	Oleanane	Oleanane	Oleanane	Oleanane	Ursane
Molecular Formula	C_30_H_48_O_5_	C_30_H_48_O_3_	C_32_H_48_O_5_	C_30_H_46_O_4_	C_30_H_48_O_4_	C_30_H_48_O_3_	C_30_H_48_O_3_
Molecular Weight (g/mol)	488.7	456.7	512.7	470.68	472.7	456.7	456.7
Key Functional Groups	3 OH (C2, C3, C23), COOH at C28	OH at C3, COOH at C28	Acetyl at C3, 11-keto, COOH at C28	OH at C3, COOH at C30	Diol at C2, C3, COOH at C28	OH at C3, COOH at C28	OH at C3, COOH at C28
Double Bond Location	Δ^12^(13)	C20=C29 (exocyclic)	Δ^12^(13)	Δ^12^(13)	Δ^12^(13)	Δ^12^(13)	Δ^12^(13)
XLOGP3-AA	5.7	8.2	7.2	5.5	6.5	7.5	7.3
Topological Polar Surface Area (Å^2^)	98	57.5	80.7	~75	77.8	57.5	57.5

Footnote. Data obtained from PubChem (https://pubchem.ncbi.nlm.nih.gov/) accessed on 2 December 2025.

**Table 2:** Primary neuroprotective mechanisms of PTAs in AD.

Compound	Type of Study	Expt Model	Primary AD Mechanism	Secondary Neuroprotective Effects	Dominant Therapeutic Category	References
Asiatic acid (AA)	in vitro, in vivo clinical	SH-SY5Y cells B103 cells *Centella asiatica* aqueous extract (clinical) Phase I randomized trial (ongoing)	Mitochondrial protection and attenuation of Aβ toxicity	Antioxidant activity, pCREB-mediated synaptic signaling, neuroprotection	Mitochondrial function protection	[27,28,29,30,31,34,37,38,41,42,43]
Betulinic acid (BTA)	in vitro, in vivo	Aβ-, STZ-induced rats, SH-SY5Y cells	Reduction in Aβ oligomerization and enhancement of proteasomal activity	Anti-inflammatory, antioxidant, anti-AChE effects	Aβ aggregation/ proteostasis regulator	[56,57,58,60,61,62,65,66,67,68,69,70,71]
Boswellic acid (BA/AKBA)	in vitro, in vivo, clinical	LPS/APP mice AD rats K-Vie™ (clinical)	Suppression of NF-κB-mediated neuroinflammation Nrf2/HO-1 antioxidant activation	Reduction in Aβ burden, attenuation of tau toxicity, neurotrophic support (BDNF), anti-AChE activity, improved cognition	Anti-inflammatory regulator	[72,74,75,77,78,79,81,85,86,87,88,89,90,92,96,98,99,100,101,102]
Glycyrrhetinic acid (GA)	in vitro, in vivo	Tauopathy mice, SH-SY5Y, *C. elegans*	Reduction in tau oligomerization; HMGB1/NF-κB-mediated neuroinflammation suppression; BACE1 inhibition; enhancement of proteasomal clearance	Antioxidant activity, cognitive improvement	Tau/proteostasis modulator	[106,108,110,111,112,115,117,120,121,122,123,124,125,126]
Maslinic acid (MA)	in vitro, in vivo	PC12 cells Scopolamine-treated mice	Suppression of RAGE/NF-κB/MAPK signaling; modulation of GSK3β-associated tau pathology	TrkB/BDNF/CREB neurotrophic signaling, glutamate homeostasis (GLAST/GLT-1), antioxidant and anti-inflammatory activity, anti-AChE activity	Tau/ neuroinflammatory modulator	[137,139,142,143,146]
Oleanolic acid (OA)	in vitro, in vivo	SH-SY5Y cells N2a/APP695swe cells	BACE1-mediated reduction in Aβ production; autophagy activation; ferroptosis suppression	Antioxidant activity, anti-inflammatory effects, mitochondrial protection, CREB/BDNF-mediated neurotrophic support, anti-AChE activity	Amyloidogenesis/ autophagy regulator	[159,160,161,163,165,166,167,168,169]
Ursolic acid (UA)	in vitro, in vivo	PC12 cells, Aβ_25–35_-induced mice, AlCl_3_-treated rats	BACE1 inhibition; enhancement of proteostasis/proteasomal activity	Antioxidant effects, anti-inflammatory activity, neurogenesis and cognitive improvement, anti-AChE activity	Amyloidogenesis/ proteostasis regulator	[170,185,186,188,189,190,191,194]
Arjunolic acid (ARA)	in vivo (non-AD models)	LPS mice, cerebral I/R rats	Activation of SIRT1/AMPK/Notch1 survival signaling	Antioxidant and anti-inflammatory effects	Neuroprotective signaling modulator	[196,197]
Corosolic acid (CA)	in silico in vivo (5xFAD)	5xFAD mice (diAcCA), Docking on tau	Inhibition of Aβ and tau aggregation	Synaptic protection and anti-inflammatory effects	Aggregation inhibitor	[198,199]
Madecassic acid (MAA)	in vitro, in vivo	SH-SY5Y cells 5xFAD mice (CWA)	Reduction in Aβ burden and mitochondrial regulation	Antioxidant and anti-AChE effects	Mitochondrial/ amyloid modulator	[11,202,205]

Abbreviations. Aβ: Amyloid-beta; AChE: Acetylcholinesterase; AKBA: Acetyl-11-keto-β-boswellic acid; AMPK: AMP-activated protein kinase; APP: Amyloid precursor protein; BACE1: Beta secretase-1; BDNF: Brain-derived neurotrophic factor; CAW: *C. asiatica* water extract; CREB: cAMP response element-binding protein; DiAcCA: di-acetylated corsolic acid; ER: Endoplasmic reticulum; GLAST: Glutamate-aspartate transporter; GLT-1:Glutamate transporter-1; GLUT: Glucose Transporter; HO-1: Heme oxygenase 1; I/R: Ischemia/reperfusion injury; LPS: Lipopolysaccharide; NF-κB: Nuclear factor kappa light-chain enhancer of activated B cells; Nrf2: Nuclear factor erythroid 2-related factor 2; RAGE: Receptor for advanced glycation end-products; ROS: Reactive oxygen species; SIRT: Sirtuin; TrkB: Tropomyosin receptor kinase.

## Data Availability

The original contributions presented in this study were included in the article.
